# Repetitive transcranial magnetic stimulation for vestibular migraine in women of reproductive age: a retrospective propensity score-matched study

**DOI:** 10.3389/fneur.2025.1735437

**Published:** 2026-01-05

**Authors:** Jianjian Zhu, Hongmei Fan, Xiao Ma, Yimin Fan, Qingjie Mao, Jing Xiao, Xiulan Xu, Beihai Ge

**Affiliations:** 1 Department of Neurology, The People's Hospital of Rugao, Nantong, Jiangsu, China; 2 Department of Otolaryngology, The People's Hospital of Rugao, Nantong, Jiangsu, China; 3 Department of Neurology, Yangxian People's Hospital, Hanzhong, Shaanxi, China

**Keywords:** neuromodulation therapy, propensity score matching, repetitive transcranial magnetic stimulation, vestibular migraine, women of reproductive age

## Abstract

**Background:**

Vestibular migraine (VM) substantially impairs quality of life, particularly in women of reproductive age. While repetitive transcranial magnetic stimulation (rTMS) has emerged as a promising non-invasive neuromodulation therapy, its efficacy for VM remains underexplored.

**Methods:**

In this retrospective study, we analyzed data from 83 women of reproductive age diagnosed with VM who were treated between June 2022 and October 2024. After propensity score matching, 34 patients who received rTMS combined with pharmacotherapy were compared with 34 patients who received pharmacotherapy alone.

**Results:**

At the 3-month follow-up, the rTMS group demonstrated a significant reduction in headache-related impact compared to the control group, as measured by HIT-6 (46.50 [43.00, 52.50] vs. 53.50 [50.00, 58.75], P-holm < 0.001), with a moderate effect size (r = 0.49, 95% CI: 0.31, 0.67). In contrast, no significant between-group differences were observed in pain intensity (VAS: 4.00 [3.00, 4.00] vs. 4.00 [4.00, 5.00], P-holm = 0.054) or dizziness handicap (DHI-T: 23.82 ± 3.39 vs. 25.88 ± 4.26, P-holm = 0.09). Within-group analyses revealed that the rTMS group exhibited continued improvement over time across both pain and vertigo measures.

**Conclusion:**

These findings indicate that as an adjunct to pharmacotherapy, rTMS offers significant benefits in alleviating pain-related functional impairment over a 3-month period in women of reproductive age with VM, although it confers no additional advantage over pharmacotherapy alone in reducing pain intensity or vertigo symptoms.

## 1 Introduction

Vestibular migraine (VM) is a neurological disorder characterized by recurrent episodes of vertigo and moderate-to-severe headache, and it is particularly prevalent among women of reproductive age (1), with an estimated prevalence of approximately 1–3% (2). VM attacks are not only associated with intense headaches but are also frequently accompanied by nausea, vomiting, photophobia, and vestibular dysfunction—including spontaneous, positional, visually triggered, or head-motion-induced vertigo—all of which significantly impair patients’ quality of life and ability to perform daily activities (3).

Pharmacological therapies, including beta-blockers, calcium channel blockers, antiepileptics, and tricyclic antidepressants, represent the first-line preventive strategy for VM (4, 5). However, many patients fail to respond adequately, experience intolerable side effects, or suffer from frequent recurrences, highlighting a significant unmet clinical need. In this context, anti-calcitonin gene-related peptide (anti-CGRP) monoclonal antibodies (e.g., erenumab, galcanezumab) and small-molecule receptor antagonists (gepants) have emerged as promising alternatives. These agents selectively block CGRP binding to its receptors, thereby inhibiting neurogenic inflammation, vasodilation, and sensitization of vestibular nuclei—mechanisms that contribute to reducing the frequency of vertigo attacks and headache in VM (6, 7). Observational studies indicate that anti-CGRP therapy significantly decreases the monthly number of vertigo days, Dizziness Handicap Inventory (DHI) scores, and improves quality of life, with a favorable tolerability profile (8, 9). Its advantages include high target specificity and a low systemic side-effect burden, particularly suiting patients with inadequate response to conventional medications or cardiovascular contraindications. However, current evidence predominantly stems from retrospective and small prospective studies, lacking support from large-scale randomized controlled trials. Furthermore, high costs and limited insurance coverage substantially restrict its clinical accessibility, especially in low- and middle-income countries (10, 11). This underscores the necessity to explore non-pharmacological, cost-effective alternative or adjunctive approaches, such as repetitive transcranial magnetic stimulation (rTMS).

In recent years, repetitive transcranial magnetic stimulation (rTMS), a non-invasive neuromodulation technique, has demonstrated therapeutic potential for migraine by regulating cortical excitability, with studies indicating reductions in headache frequency, severity, and duration (6, 7). The primary visual cortex (V1) may represent a central locus in the pathophysiology of migraine with aura. Neuroimaging evidence reveals that migraine patients exhibit enhanced low-frequency oscillations and increased functional connectivity between V1 and other sensory cortices during the interictal phase, suggesting a neural substrate for visual hypersensitivity (12). Complementary animal studies confirm that altered excitability and synaptic transmission in V1 elevate visually evoked gamma oscillations, underscoring a critical excitatory-inhibitory imbalance (13, 14). Given this pathological basis of occipital hyperexcitability, targeted neuromodulation has emerged as a direct intervention. Single-pulse TMS (sTMS) applied to the occipital lobe suppresses cortical excitability via enhanced GABAergic inhibition, a mechanism that supports its FDA approval for the acute treatment of migraine with aura (15–17). Similarly, low-frequency rTMS delivered to the occipital region can alleviate symptoms by modulating functional connectivity within visual networks (18). In contrast to these advances, the application of rTMS in VM remains underexplored and warrants systematic evaluation.

The pathophysiology of VM involves both functional and structural alterations in brain networks integral to multisensory integration. Key among these are aberrant connectivity within the vestibulo-pain network and increased gray matter volume in the occipital lobe, which have been associated with deficits in visual-vestibular processing (19). Electrophysiological evidence further supports visual system dysregulation, with VM patients showing reduced P1 amplitudes and shorter latencies to visual stimuli during the interictal period, indicating abnormal cortical activation and sensory processing (20). At the network level, functional MRI and dynamic functional network connectivity (dFNC) analyses demonstrate enhanced coupling between the extrastriate visual network (eVN) and higher-order cognitive networks—including the ventral attention network (VAN), default mode network (DMN), and left frontoparietal network (lFPN)—together with weakened connectivity between visual and auditory networks (AuN) (21). These aberrant cross-network interactions are thought to promote visual cortical hyperexcitability and impaired multisensory integration, which may facilitate VM onset. During acute episodes, visually driven neural activity likely engages the temporo-parieto-occipital junction and vestibular nuclei, heightening cortical sensitivity to visual motion and thereby eliciting visual aura and headache symptoms (22). Collectively, these results point to visual cortical hyperexcitability and dysregulated interactions between vestibular and cognitive networks as a neural basis of key symptoms in VM. In this context, the ability of rTMS to induce long-term modulation of visual network function and restore inter-network balance offers a promising therapeutic avenue—one that warrants further systematic investigation.

Current assessments of VM treatment outcomes include measures of headache intensity (e.g., Visual Analogue Scale [VAS], Headache Impact Test [HIT-6]), disability (e.g., Migraine Disability Assessment [MIDAS]), and vestibular-related dysfunction (e.g., Dizziness Handicap Inventory [DHI]). Studies have demonstrated that HIT-6 and MIDAS exhibit good validity in evaluating headache-related quality of life and disability (23), while DHI shows strong reliability in assessing dizziness and balance impairment in VM (24).

In the present study, we retrospectively analyzed data from 83 women of reproductive age diagnosed with VM who received either rTMS combined with pharmacotherapy or pharmacotherapy alone. Propensity score matching (PSM) was applied to control for baseline confounding factors, enabling a comprehensive evaluation of the short-term (2 weeks) and medium-term (3 months) efficacy of rTMS across multiple outcome dimensions, including VAS, HIT-6, and DHI. We hypothesized that rTMS combined with pharmacotherapy would yield superior improvements in headache and vertigo symptoms compared to pharmacotherapy alone, and that effect size analyses would further clarify its clinical significance. The findings of this study are expected to provide evidence-based support for non-pharmacological interventions in VM and to inform the development of personalized treatment strategies.

## 2 Materials and methods

### 2.1 Study design and population

This was a single-center, retrospective cohort study. Data were obtained from the electronic medical record (EMR) system of the People’s Hospital of Rugao. We enrolled female patients with VM who attended the neurology or otolaryngology outpatient clinics of the People’s Hospital of Rugao between June 2022 and October 2024. The study was approved by the Ethics Committee of the People’s Hospital of Rugao (approval number: KY202206001).

The participants in this study were women of reproductive age, diagnosed with VM according to the diagnostic criteria for VM (25). They were divided into two groups based on treatment regimen: the rTMS combined with pharmacotherapy group (rTMS group) and the pharmacotherapy alone group (control group). Baseline demographic and clinical data—including age, duration of headache attacks, and vestibular symptoms—were collected.

Inclusion Criteria: Diagnosis of VM (definite or probable VM) based on established criteria: women of reproductive age (20–50 years); disease duration ≥3 months; no prior history of TMS; no regular use of nonsteroidal anti-inflammatory drugs; preventive antimigraine medications, or antipsychotic drugs within the past 6 months; complete outpatient medical records.

Exclusion Criteria: History of epilepsy or febrile seizures; history of cerebrovascular disease, intracranial space-occupying lesions, intracranial tumors, or psychiatric disorders; diagnoses of Ménière’s disease, benign paroxysmal positional vertigo, or vestibular neuritis; presence of metallic implants, such as cardiac pacemakers, cochlear implants, carotid artery stents, or intracranial coils; confirmed patent foramen ovale via transthoracic echocardiography and right heart function assessment; women with plans for pregnancy within 6 months; and patients with missing data exceeding 20%.

### 2.2 Data collection

Data were collected by professionally trained neurologists and otolaryngologists. The collected data included: (1) demographic characteristics and clinical symptoms, such as age, headache duration, and vestibular symptoms; and (2) standardized scale scores (VAS, HIT-6, and DHI). The DHI scores encompassed the total score (DHI-T) and subscale scores for physical (DHI-P), emotional (DHI-E), and functional (DHI-F) impairment. All scale assessments were conducted at baseline, 2 weeks post-treatment, and 3 months post-treatment.

### 2.3 Treatment protocol

The treatment protocol consisted of (1) pharmacological therapy and (2) rTMS therapy. All patients received pharmacological treatment, which included non-steroidal anti-inflammatory analgesics (ibuprofen or diclofenac sodium) for acute headache attacks, along with a three-month preventive regimen of anti-vertigo medications (flunarizine 5 mg once nightly and betahistine 6 mg three times daily). Guided by the pathological role of visual cortical hyperexcitability in VM, V1 was selected as the stimulation target. Low-frequency (1 Hz) rTMS was applied to induce long-term depression (LTD) and thereby reduce cortical excitability. Stimulation was delivered using a CCY-I magnetic stimulator (Yiruide, Wuhan, China) equipped with a figure-of-eight coil (inner wing diameter: 7.5 cm). The coil was positioned tangentially on the scalp, with the handle oriented upward and perpendicular to the sagittal plane. The stimulation site was located over the occipital cortex, 2 cm posterior to the Oz electrode according to the 10–20 electroencephalography (EEG) system, corresponding to the primary visual cortex (V1). Stimulation parameters were set at 1 Hz and 90% of the resting motor threshold (RMT). Each 20 min session consisted of 10 s trains separated by 2 s inter-train intervals, delivering a total of 1,000 pulses per session. Treatment was administered 5 days per week for 2 weeks (cumulative dose: 10,000 pulses), followed by a three-month follow-up period.

### 2.4 Statistical analysis

All statistical analyses were performed using R software (version 4.3.2). The distribution of continuous variables was assessed for normality using the Shapiro–Wilk test. Based on this, continuous data are summarized as Mean ± SD or Median (Q1, Q3). Between-group differences for these variables were evaluated using independent-samples *t*-tests (for normally distributed data) or Mann–Whitney U tests (for non-normally distributed data), with effect sizes quantified as Cohen’s d (for *t*-tests) or r (for U tests). Within-group changes from baseline were analyzed using paired-samples *t*-tests or Wilcoxon signed-rank tests, with corresponding effect sizes (Cohen’s d or r). Multiple comparisons across the three time points for both between-group and within-group outcomes were adjusted using the Holm method. Categorical variables, expressed as counts (percentages), were compared between groups using Pearson’s chi-square or Fisher’s exact test. All reported *p*-values are two-tailed, and statistical significance was defined as *p* < 0.05. Effect sizes are presented with their 95% confidence intervals where applicable.

To minimize potential confounding effects, PSM was employed to balance baseline characteristics between the groups. The propensity score was estimated using a logistic regression model with group assignment as the dependent variable and all measured baseline characteristics as covariates. A 1:1 nearest-neighbor matching algorithm was implemented without replacement, using a caliper width of 0.2 standard deviations of the logit of the propensity score. Covariate balance between the matched groups was assessed using standardized mean differences (SMD), with an SMD < 0.1 indicating adequate balance.

## 3 Results

**Figure 1:**
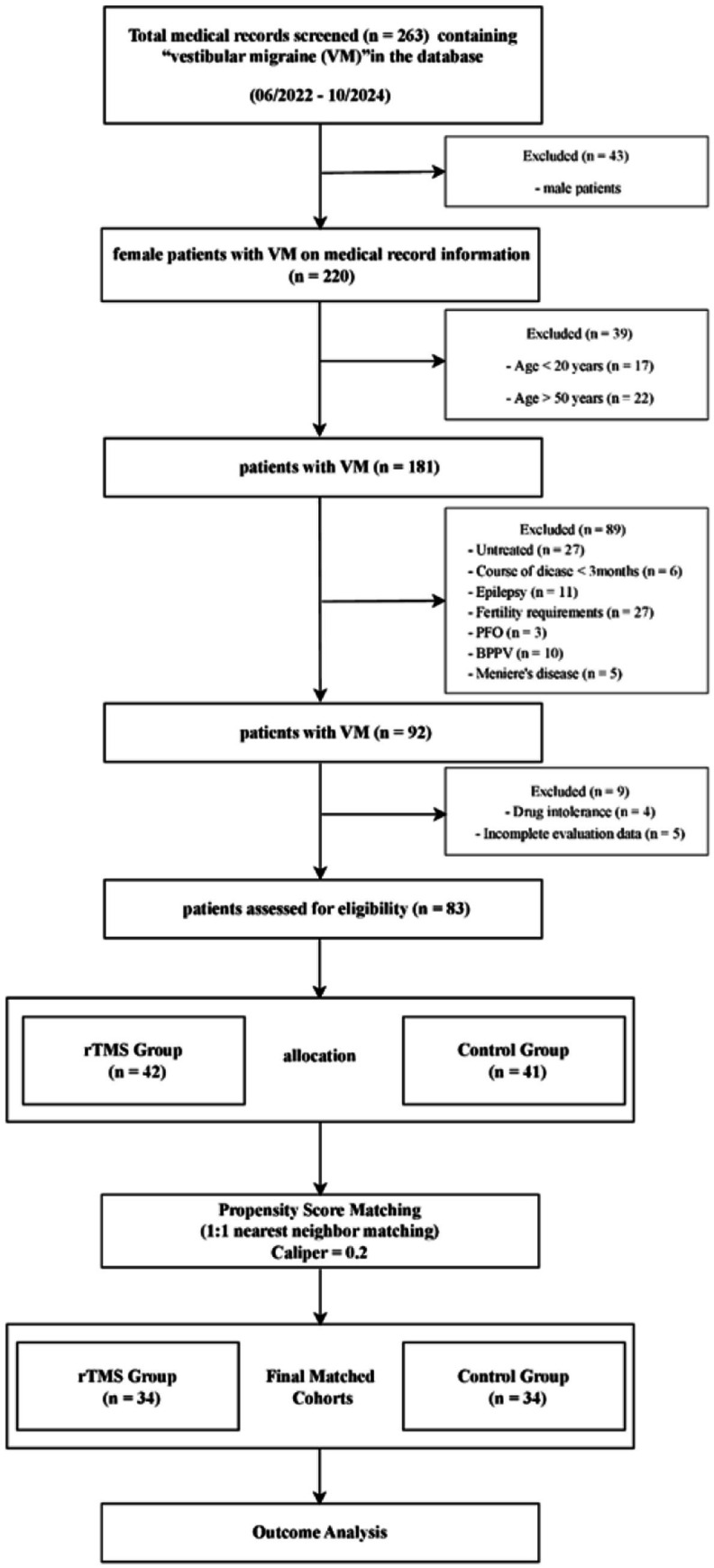
Flowchart of patient selection and propensity score matching for VM in women of reproductive age. VM, vestibular migraine; PFO, patent foramen ovale; BPPV, benign paroxysmal positional vertigo; rTMS, repetitive transcranial magnetic stimulation.

Based on the predefined inclusion and exclusion criteria, a total of 83 eligible participants were ultimately selected from an initial pool of 263 screened patients (Figure 1). These participants were divided into two groups: the rTMS combined with pharmacotherapy group (rTMS group, *n* = 42) and the pharmacotherapy alone group (control group, *n* = 41).

### 3.1 Propensity score matching

Before propensity score matching, the overall study population had a mean age of 38.06 ± 7.36 years and a mean headache attack duration of 4.33 ± 1.20 days. No significant intergroup differences were observed in baseline scores for the VAS, HIT-6, DHI-T, and its subscales (DHI-F, DHI-E, DHI-P) (all *p* > 0.05; Table 1).

**Table 1:** Baseline characteristics of patients before and after propensity score matching.

Variable	Before PSM					
	Total (*n* = 83)	Control group (*n* = 41)	rTMS group (*n* = 42)	Statistic	*P*-value	SMD
AGE, Mean ± SD	38.06 ± 7.36	38.05 ± 7.58	38.07 ± 7.23	t = 0.01	0.99	0.00
Duration of headache, Mean ± SD	4.33 ± 1.20	4.29 ± 1.08	4.36 ± 1.32	t = 0.24	0.81	0.05
VAS, M [Q1, Q3]	5.00 [4.00, 6.00]	5.00 [4.00, 6.00]	5.00 [4.00, 6.00]	Z = 0.19	0.85	0.03
HIT-6, M [Q1, Q3]	65.00 [64.00, 67.00]	65.00 [64.00, 67.00]	66.00 [64.00, 67.00]	Z = 0.98	0.33	0.16
DHI-T, M [Q1, Q3]	32.00 [30.00, 34.00]	32.00 [30.00, 34.00]	32.00 [30.00, 34.00]	Z = 0.62	0.54	0.21
DHI-E, M [Q1, Q3]	10.00 [8.00, 13.00]	10.00 [8.00, 14.00]	10.00 [8.00, 12.00]	Z = 0.20	0.85	0.09
DHI-F, M [Q1, Q3]	10.00 [9.00, 13.00]	10.00 [8.00, 12.00]	12.00 [10.00, 14.00]	Z = 1.06	0.29	0.25
DHI-P, M [Q1, Q3]	10.00 [8.00, 12.00]	10.00 [8.00, 12.00]	10.00 [8.00, 12.00]	Z = 0.53	0.60	0.11
Nausea vomiting, *n* (%)				χ^2^ = 0.01	0.91	0.07
0	42 (50.60)	20 (48.78)	22 (52.38)			
1	41 (49.40)	21 (51.22)	20 (47.62)			
Tinnitus ear fullness, *n* (%)				χ^2^ = 0.00	1.00	0.02
0	51 (61.45)	25 (60.98)	26 (61.9)			
1	32 (38.55)	16 (39.02)	16 (38.1)			
Unsteady gait, *n* (%)				χ^2^ = 0.02	0.88	0.09
0	55 (66.27)	28 (68.29)	27 (64.29)			
1	28 (33.73)	13 (31.71)	15 (35.71)			
Positional vertigo, *n* (%)				χ^2^ = 0.38	0.54	0.19
0	55 (66.27)	29 (70.73)	26 (61.90)			
1	28 (33.73)	12 (29.27)	16 (38.10)			

Variable	After PSM					
	Total (*n* = 68)	Control group (*n* = 34)	rTMS group (*n* = 34)	Statistic	*P*-value	SMD
AGE, Mean ± SD	37.53 ± 7.65	37.35 ± 7.96	37.71 ± 7.45	t = 0.19	0.85	0.05
Duration of Headache, Mean ± SD	4.35 ± 1.17	4.38 ± 1.13	4.32 ± 1.22	t = −0.21	0.84	0.05
VAS, M [Q1, Q3]	5.00 [4.00, 6.00]	5.00 [4.00, 6.00]	5.00 [4.00, 6.00]	Z = 0.20	0.84	0.03
HIT-6, M [Q1, Q3]	65.00 [64.00, 67.00]	65.00 [64.00, 67.00]	65.00 [63.00, 66.75]	Z = 0.25	0.80	0.07
DHI-T, M [Q1, Q3]	32.00 [30.00, 34.00]	32.00 [30.00, 34.00]	32.00 [30.00, 33.50]	Z = 0.22	0.83	0.07
DHI-E, M [Q1, Q3]	10.00 [8.00, 14.00]	10.00 [8.00, 14.00]	12.00 [8.00, 12.00]	Z = 0.19	0.85	0.02
DHI-F, M [Q1, Q3]	10.00 [8.00, 12.00]	10.00 [8.00, 12.00]	10.00 [8.50, 12.00]	Z = 0.10	0.92	0.05
DHI-P, M [Q1, Q3]	10.00 [8.00, 12.00]	10.00 [8.00, 12.00]	10.00 [8.00, 12.00]	Z = 0.08	0.94	0.02
Nausea vomiting, *n* (%)				χ^2^ = 0.00	1.00	0.00
0	34 (50.00)	17 (50.00)	17 (50.00)			
1	34 (50.00)	17 (50.00)	17 (50.00)			
Tinnitus ear fullness, *n* (%)				χ^2^ = 0.00	1.00	0.00
0	42 (61.76)	21 (61.76)	21 (61.76)			
1	26 (38.24)	13 (38.24)	13 (38.24)			
Unsteady gait, *n* (%)				χ^2^ = 0.00	1.00	0.00
0	44 (64.71)	22 (64.71)	22 (64.71)			
1	24 (35.29)	12 (35.29)	12 (35.29)			
Positional vertigo, *n* (%)				χ^2^ = 0.00	1.00	0.06
0	45 (66.18)	23 (67.65)	22 (64.71)			
1	23 (33.82)	11 (32.35)	12 (35.29)			

Categorical variables are presented as *n* (%); continuous variables are presented as Mean ± SD or Median [Q1, Q3] as appropriate. VAS, Visual Analogue Scale; HIT-6, Headache Impact Test; DHI, Dizziness Handicap Inventory; PSM, Propensity Score Matching; SMD, Standardized Mean Difference. An SMD value of <0.1 indicates good intergroup balance.

**Figure 2:**
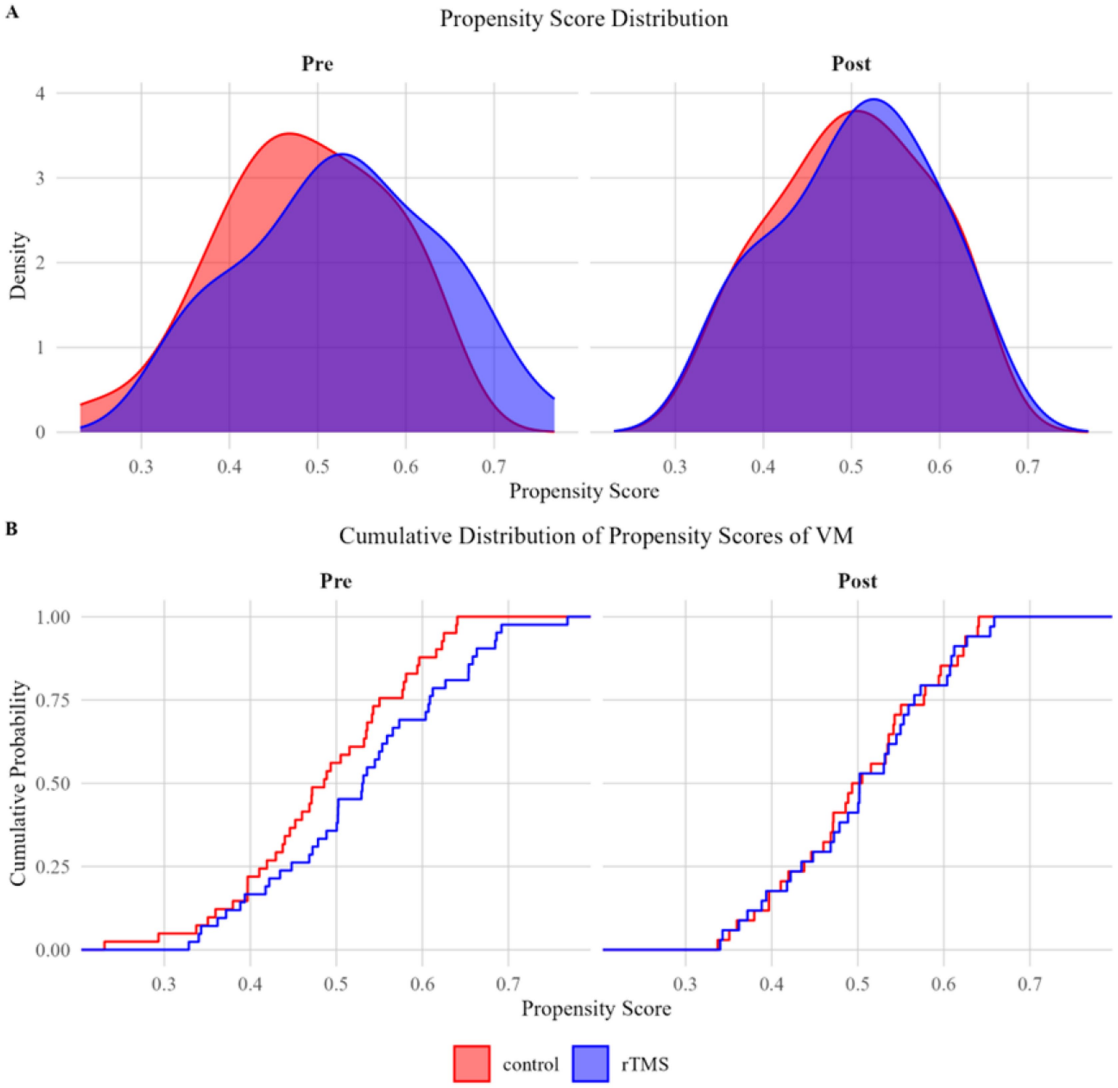
**(A, B)** Propensity score distributions before and after matching in patients with VM.

After matching, 34 patients were allocated to each group (rTMS and control). The matched cohorts remained well-balanced across all key clinical measures, including VAS, HIT-6, DHI-T, and its subscales (DHI-F, DHI-E, DHI-P), with all SMD below 0.1 (Table 1). The overall covariate balance was considered satisfactory, supporting the robustness of subsequent between-group efficacy analyses (Figure 2).

### 3.2 Between-group efficacy comparisons

#### 3.2.1 Pain-related outcomes

**Figure 3:**
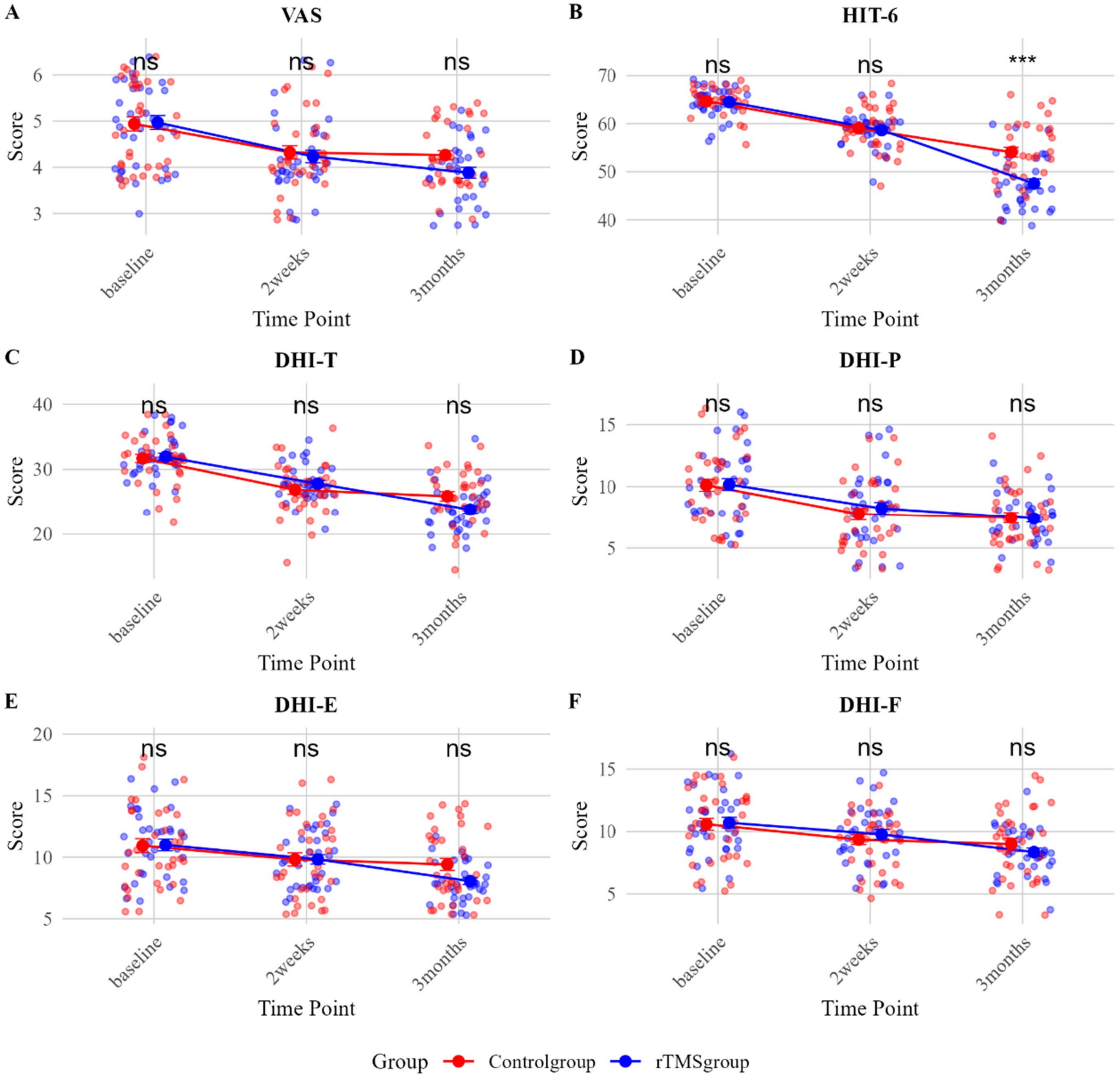
**(A–F)** Group comparisons of clinical outcomes after PSM of VM (Holm-corrected).

VAS Score: At 2 weeks, there was no significant difference between the two groups (rTMS group: 4.00 [4.00, 4.75] vs. control group: 4.00 [4.00, 5.00]; P-holm = 1.00). At 3 months, the median VAS score was lower in the rTMS group (4.00 [3.00, 4.00]) than in the control group (4.00 [4.00, 5.00]); however, this difference was not statistically significant after correction for multiple comparisons (P-holm = 0.054), with a small observed effect size (r = 0.29) (Table 2; Figure 3).

**Table 2:** Comparison of clinical outcomes between the control and rTMS groups.

Variable	Time point	Control group (*n* = 34)	rTMS group (*n* = 34)	Statistic	*P*-value (Holm-corrected)	Effect size (95%CI)
VAS	Baseline	5.00 [4.00, 6.00]	5.00 [4.00, 6.00]	Z = −0.20	1.00	r = 0.02 (−0.22, 0.26)
	2 weeks	4.00 [4.00, 5.00]	4.00 [4.00, 4.75]	Z = −0.43	1.00	r = 0.05 (−0.19, 0.29)
	3 months	4.00 [4.00, 5.00]	4.00 [3.00, 4.00]	Z = −2.37	0.054	r = 0.29 (0.07, 0.51)
HIT-6	Baseline	65.00 [64.00, 67.00]	65.00 [63.00, 66.75]	Z = −0.25	1.00	r = 0.03 (−0.21, 0.27)
	2 weeks	59.00 [56.00, 63.00]	59.50 [58.00, 60.00]	Z = −0.23	1.00	r = 0.03 (−0.21, 0.27)
	3 months	53.50 [50.00, 58.75]	46.50 [43.00, 52.50]	Z = −4.03	<0.001	r = 0.49 (0.31, 0.67)
DHI-T	Baseline	32.00 [30.00, 34.00]	32.00 [30.00, 33.50]	Z = −0.22	0.83	r = 0.03 (−0.21, 0.27)
	2 weeks	28.00 [24.00, 28.00]	28.00 [26.00, 29.50]	Z = −1.08	0.56	r = 0.13 (−0.11, 0.37)
	3 months	25.88 ± 4.26	23.82 ± 3.39	t = −2.20*	0.09	d = −0.54 (−1.02, −0.05)
DHI-P	Baseline	10.00 [8.00, 12.00]	10.00 [8.00, 12.00]	Z = −0.08	1.00	r = 0.01 (−0.23, 0.25)
	2 weeks	8.00 [6.00, 10.00]	8.00 [6.00, 10.00]	Z = −0.76	1.00	r = 0.09 (−0.15, 0.33)
	3 months	7.00 [6.00, 8.00]	8.00 [6.00, 8.00]	Z = −0.21	1.00	r = 0.03 (−0.21, 0.26)
DHI-E	Baseline	10.00 [8.00, 14.00]	12.00 [8.00, 12.00]	Z = −0.19	1.00	r = 0.02 (−0.22, 0.26)
	2 weeks	10.00 [8.00, 12.00]	10.00 [8.00, 12.00]	Z = −0.25	1.00	r = 0.03 (−0.21, 0.27)
	3 months	8.00 [8.00, 12.00]	8.00 [6.50, 9.50]	Z = −1.74	0.24	r = 0.21 (−0.02, 0.44)
DHI-F	Baseline	10.00 [8.00, 12.00]	10.00 [8.50, 12.00]	Z = −0.10	1.00	r = 0.01 (−0.23, 0.25)
	2 weeks	10.00 [8.00, 10.00]	10.00 [8.00, 12.00]	Z = −0.49	1.00	r = 0.06 (−0.18, 0.30)
	3 months	10.00 [6.50, 10.00]	8.00 [8.00, 10.00]	Z = −1.17	0.73	r = 0.14 (−0.09, 0.38)

* Data are normally distributed. *P*-values were adjusted for multiple comparisons using the Holm method. Effect sizes are presented as Cohen’s d for parametric tests or r (Z/√N) for non-parametric tests, with 95% confidence intervals.

HIT-6 Score: At 2 weeks, there was no significant difference between the two groups (59.50 [58.00, 60.00] vs. 59.00 [56.00, 63.00], P-holm = 1.00). At 3 months, HIT-6 scores were significantly lower in the rTMS group (46.50 [43.00, 52.50]) compared to the control group (53.50 [50.00, 58.75]; P-holm < 0.001), with a moderate effect size (r = 0.49, 95% CI: 0.31, 0.67). These results indicate that rTMS combined with pharmacotherapy was superior to pharmacotherapy alone in reducing headache-related functional impact (Table 2; Figure 3).

#### 3.2.2 Vertigo-related outcomes

DHI-T: At 2 weeks, there was no significant difference between the two groups (28.00 [26.00, 29.50] vs. 28.00 [24.00, 28.00], P-holm = 0.56). At 3 months, the DHI-T score was numerically lower in the rTMS group (23.82 ± 3.39) than in the control group (25.88 ± 4.26); however, this difference was not statistically significant after Holm correction for multiple comparisons (P-holm = 0.09). Furthermore, none of the DHI subscales (DHI-P, DHI-E, DHI-F) showed any significant between-group differences at any assessed time point following the same correction (all P-holm >0.05) (Table 2; Figure 3).

### 3.3 Within-group efficacy comparisons

#### 3.3.1 Pain-related indicators

**Figure 4:**
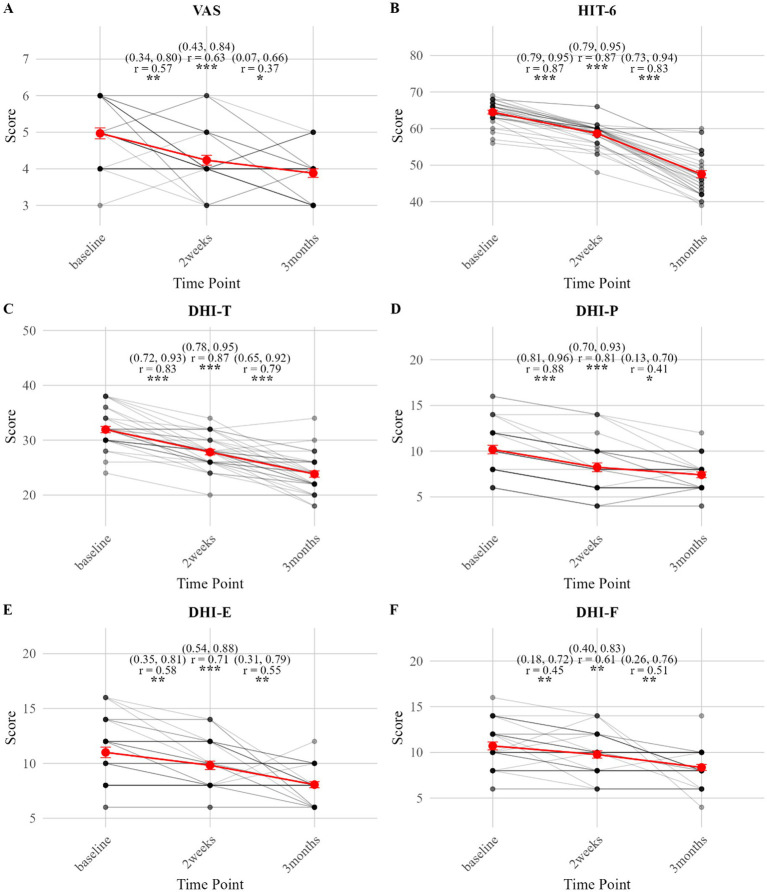
**(A–F)** Longitudinal changes in clinical outcomes in VM patients receiving rTMS treatment (effect sizes with 95% CIs).

In the rTMS group, both VAS and HIT-6 scores decreased significantly from baseline at both the 2-week and 3-month follow-ups (all P-holm < 0.05), with effect sizes ranging from moderate to large. From 2 weeks to 3 months, VAS scores showed a further significant reduction (P-holm = 0.03; with a moderate effect size: r = 0.37, 95% CI: 0.07, 0.66). HIT-6 scores also continued to improve significantly during this period (P-holm <0.001; with a large effect size: r = 0.83, 95% CI: 0.73, 0.93) (Table 3; Figure 4).

**Table 3:** Longitudinal comparisons of clinical outcomes within the control and rTMS groups.

Variable	Comparison	Control group (*n* = 34)			rTMS group (*n* = 34)		
		Statistic	*P*-value (Holm-corrected)	Effect size (95%CI)	Statistic	*P*-value (Holm-corrected)	Effect size (95%CI)
VAS	Baseline vs. 2 weeks	Z = 2.65	0.02	r = 0.45 (0.18, 0.72)	Z = 3.33	<0.001	r = 0.57 (0.34, 0.80)
	Baseline vs. 3 months	Z = 3.29	<0.01	r = 0.55 (0.32, 0.79)	Z = 3.70	<0.01	r = 0.63 (0.43, 0.84)
	2 weeks vs. 3 months	Z = 0.34	0.74	r = 0.06 (−0.28, 0.40)	Z = 2.13	0.03	r = 0.37 (0.07, 0.66)
HIT-6	Baseline vs. 2 weeks	Z = 4.86	<0.001	r = 0.83 (0.73, 0.94)	Z = 5.09	<0.001	r = 0.87 (0.79, 0.95)
	Baseline vs. 3 months	Z = 5.08	<0.001	r = 0.87 (0.79, 0.95)	Z = 5.08	<0.001	r = 0.87 (0.79, 0.95)
	2 weeks vs. 3 months	Z = 4.22	<0.001	r = 0.72 (0.56, 0.89)	Z = 4.86	<0.001	r = 0.83 (0.73, 0.94)
DHI-T	Baseline vs. 2 weeks	Z = 5.06	<0.001	r = 0.87 (0.78, 0.95)	Z = 4.81	<0.001	r = 0.83 (0.72, 0.93)
	Baseline vs. 3 months	Z = 4.96	<0.001	r = 0.85 (0.76, 0.95)	Z = 5.05	<0.001	r = 0.87 (0.78, 0.95)
	2 weeks vs. 3 months	Z = 1.66	0.10	r = 0.29 (−0.03, 0.60)	Z = 4.58	<0.001	r = 0.79 (0.65, 0.92)
DHI-P	Baseline vs. 2 weeks	Z = 5.50	<0.001	r = 0.94 (0.91, 0.98)	Z = 5.15	<0.001	r = 0.88 (0.81, 0.96)
	Baseline vs. 3 months	Z = 4.95	<0.001	r = 0.85 (0.75, 0.94)	Z = 4.74	<0.001	r = 0.81 (0.70, 0.93)
	2 weeks vs. 3 months	Z = 0.93	0.35	r = 0.16 (−0.17, 0.49)	Z = 2.41	0.02	r = 0.41 (0.13, 0.70)
DHI-E	Baseline vs. 2 weeks	Z = 2.41	0.03	r = 0.42 (0.13, 0.70)	Z = 3.37	<0.01	r = 0.58 (0.35, 0.81)
	Baseline vs. 3 months	Z = 3.09	<0.01	r = 0.53 (0.29, 0.78)	Z = 4.13	<0.001	r = 0.71 (0.54, 0.88)
	2 weeks vs. 3 months	Z = 1.25	0.21	r = 0.21 (−0.11, 0.54)	Z = 3.29	<0.01	r = 0.55 (0.31, 0.79)
DHI-F	Baseline vs. 2 weeks	Z = 3.85	<0.001	r = 0.66 (0.47, 0.85)	Z = 2.61	<0.01	r = 0.45 (0.18, 0.72)
	Baseline vs. 3 months	Z = 3.80	<0.001	r = 0.65 (0.45, 0.85)	Z = 3.56	<0.01	r = 0.61 (0.40, 0.83)
	2 weeks vs. 3 months	Z = 1.32	0.19	r = 0.23 (−0.10, 0.55)	Z = 2.97	<0.01	r = 0.51 (0.26, 0.76)

*P*-values were adjusted for multiple comparisons using the Holm method. Effect sizes are presented as Cohen’s d for parametric tests or r (Z/√N) for non-parametric tests, with 95% confidence intervals.

**Figure 5:**
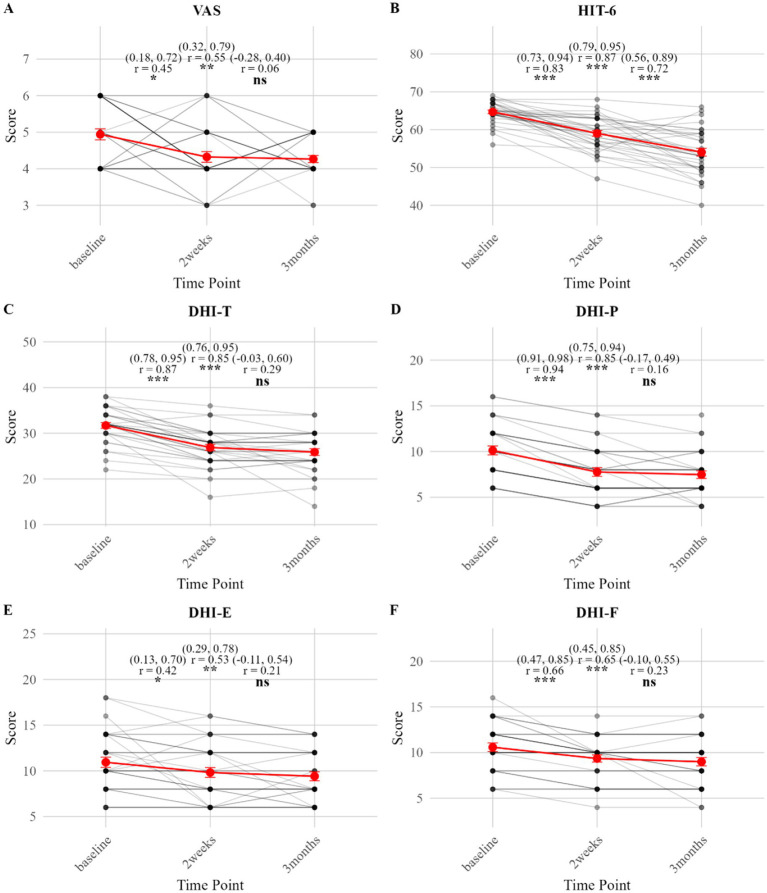
**(A–F)** Longitudinal changes in clinical outcomes in VM patients receiving control treatment (effect sizes with 95% CIs).

In the control group, both VAS and HIT-6 scores also decreased significantly from baseline at both the 2-week and 3-month follow-ups (all P-holm <0.05). From 2 weeks to 3 months, VAS scores showed no significant change (P-holm = 0.74), whereas HIT-6 scores continued to improve significantly (P-holm <0.001; with a large effect size: r = 0.72, 95% CI: 0.56, 0.89) (Table 3; Figure 5).

#### 3.3.2 Vertigo-related indicators

In the rTMS group, the DHI-T score decreased significantly from baseline at both the 2-week and 3-month follow-ups (P-holm < 0.001), with large effect sizes (r ≥ 0.83). A further significant reduction in DHI-T was observed from 2 weeks to 3 months (P-holm < 0.001; with a large effect size: r = 0.79, 95% CI: 0.65, 0.92). All DHI subscales (DHI-P, DHI-E, DHI-F) showed significant decreases across all time points (all P-holm < 0.05), with moderate to large effect sizes (Table 3; Figure 4).

In the control group, the DHI-T score and all subscales decreased significantly from baseline at both the 2-week and 3-month follow-ups (all P-holm < 0.05), with moderate to large effect sizes. No significant changes in DHI-T or its subscales were observed from 2 weeks to 3 months (all P-holm >0.05) (Table 3; Figure 5).

### 3.4 Safety comparison

No significant difference in overall adverse event incidence was observed between the groups. In the rTMS group (*n* = 34), no serious adverse events were reported; only mild transient events occurred, including dizziness (*n* = 2) and fatigue (*n* = 2). Similarly, the control group (*n* = 34) reported only mild adverse events, consisting of dizziness (*n* = 1) and fatigue (*n* = 3).

## 4 Discussion

This study retrospectively analyzed the efficacy differences between rTMS combined with pharmacotherapy and pharmacotherapy alone in women of reproductive age with VM, using PSM to control for baseline confounding. Over the 3-month follow-up period, rTMS combined with medication demonstrated sustained superiority over pharmacotherapy alone in improving headache-related quality of life (HIT-6), although no significant intergroup differences were observed in overall reductions of pain intensity (VAS) or dizziness-related handicap (DHI). Notably, longitudinal assessment revealed that the rTMS group exhibited continued improvements across VAS, HIT-6, and DHI scores from 2 week to 3 months, whereas the pharmacotherapy-alone group showed no further improvement after the initial treatment phase. These findings provide clinical support for integrating rTMS into a multimodal treatment strategy for VM.

From the perspective of improvement in pain symptoms, patients with VM are more prone to experiencing occipital pain compared to those with migraine without vestibular symptoms (26). This study demonstrates that rTMS provided sustained benefit with a moderate effect size in reducing the impact of headache on quality of life, as assessed by the HIT-6 questionnaire. Even after applying a more stringent matching protocol (caliper width adjusted to 0.1), the rTMS group maintained a statistically significant and persistent advantage in HIT-6 improvement over the control group (Supplementary Table S2), with both groups showing significant reductions in HIT-6 scores from baseline. In contrast, pain intensity as measured by the VAS did not show a significant improvement. This discrepancy suggests that rTMS may have a more pronounced effect on improving functional outcomes and quality of life than on reducing pure pain intensity. Regarding its mechanisms, rTMS is thought to exert therapeutic effects through multiple neuromodulatory pathways. Meta-analyses have suggested that rTMS can modulate cortical excitability and restore the balance between inhibitory and excitatory neurotransmitters, thereby reversing central sensitization associated with chronic pain states (27). Furthermore, low-frequency rTMS has been shown to induce LTD in the hyperexcitable occipital cortex, thereby reducing abnormal inputs to the vestibular nuclei (28). Notably, the time-dependent, cumulative improvement observed in the rTMS group in the present study suggests that its therapeutic mechanism may involve synaptic plasticity changes, including LTD. This mechanism offers a plausible explanation for the sustained therapeutic effects of rTMS in VM patients.

The absence of significant intergroup differences in the DHI, despite clear within-group improvement following rTMS, points to a complex underlying mechanism. The pathophysiology of VM involves cross-activation of the vestibular-trigeminal network and abnormal cortical regulation (29). Neuroimaging findings provide further insights: functional magnetic resonance imaging (fMRI) studies in VM have revealed structural and functional alterations in the occipital lobe, including increased gray matter volume (19), as well as aberrant neural activity, including elevated amplitude of low-frequency fluctuation (ALFF) in the right lingual gyrus (30) alongside reduced ALFF in the middle and superior occipital gyri (31). Additionally, studies have reported significantly decreased regional homogeneity in the left middle occipital gyrus (32), increased FC between the left cuneus and right lingual gyrus (33), and enhanced functional network connectivity (FNC) between the visual network and the executive control network (34). This collective evidence provides a strong rationale for employing low-frequency rTMS to modulate occipital excitability. The partial treatment response may be attributed to the heterogeneous nature of cortical excitability patterns within the occipital lobe, suggesting that future studies should aim to identify patient subtypes most likely to benefit from this neuromodulatory approach.

Although numerical differences favored the rTMS group for VAS and DHI-T at 3 months, these were not statistically significant after multiplicity correction. This suggests that the additional benefit of rTMS over pharmacotherapy in reducing pure pain intensity and vertigo-related disability is limited or inconsistent. However, its benefit in improving headache-related functional impact (HIT-6) was clear and robust.

Another finding of this study was the treatment duration effect. The rTMS group demonstrated continuous improvement from 2 weeks to 3 months, whereas the pharmacotherapy group showed predominantly early-phase improvement. This phenomenon is consistent with the hypothesis that rTMS may induce delayed but sustained therapeutic effects through neuroplastic changes. Randomized trials have similarly observed cumulative benefits of rTMS in chronic migraine treatment over time (35). This time-effect pattern carries important implications for clinical treatment strategies. Neuromodulation therapies may require sufficiently long treatment durations to achieve maximal efficacy, and short-term evaluations may underestimate their potential benefits.

From a methodological perspective, this study employed PSM to address confounding biases in retrospective data, achieving optimal baseline balance between groups (SMD < 0.1). This approach holds significant value in observational studies, particularly in domains where randomized controlled trials are challenging to implement (e.g., neuromodulation therapies), as it provides higher-level evidence. Nevertheless, a key limitation inherent to the retrospective design is that PSM cannot account for unmeasured or unrecorded confounders. Specifically, several clinically relevant modifiers of VM—including hormonal status, menstrual cycle phase, psychiatric comorbidities (e.g., anxiety or depression), and sleep disturbances—were not systematically recorded in our dataset and therefore could not be adjusted for in the model. Residual imbalances in these factors may have influenced the outcome assessment.

### 4.1 Limitations and future directions

This study has several limitations inherent to its retrospective design. First, the generalizability of our findings is limited by the single-center design and moderate cohort size (*n* = 68). Statistical power for subgroup analyses or detecting modest effect sizes was further diminished by the lack of data on potential confounders such as hormonal status, psychiatric comorbidities, and sleep disorders. Second, the 3-month follow-up period, while indicative of short-term efficacy, is insufficient to determine the long-term persistence of the rTMS benefits. Longer-term observational data are needed to compare these results with the known sustainability of other interventions for VM (36). Third, the use of a fixed rTMS protocol represents a methodological constraint, as existing evidence underscores that parameters such as stimulation target, frequency, and treatment duration are critical moderators of treatment outcomes (27). Finally, our propensity score-based analysis highlighted a clear trade-off between matching precision and statistical power-a common challenge in observational studies that impacts the precision of effect estimates. Future large-scale, multicenter, prospective studies are imperative to validate the long-term efficacy of rTMS in women of reproductive age with VM, to explore personalized stimulation strategies guided by neuroimaging biomarkers, and to generate more robust evidence for clinical application.

## 5 Conclusion

This study provides preliminary evidence for the efficacy of rTMS as an adjunctive therapy in women of reproductive age with VM, highlighting its specific advantages in alleviating pain-related disability. Additionally, the synergistic effects of rTMS with other non-pharmacological interventions — including non-invasive vagus nerve stimulation (37), external trigeminal nerve stimulation (38), acupuncture (39), and resistive exercise (40)—warrant investigation to establish a comprehensive management strategy for VM.

## Data Availability

The raw data supporting the conclusions of this article will be made available by the authors, without undue reservation.
