# Supplementary material for: Repetitive transcranial magnetic stimulation for vestibular migraine in women of reproductive age: a retrospective propensity score-matched study
Source: Front Neurol. 2026 Jan 5;16:1735437. doi: 10.3389/fneur.2025.1735437 (PMC12812562; doi:10.3389/fneur.2025.1735437)
Supplement: Supplementary file 1 [file Data_Sheet_1.docx]

**Supplementary Material 1: Sensitivity Analysis with a Propensity Score Matching Caliper of 0.1**

A sensitivity analysis was performed using a stricter propensity score matching (PSM) caliper width of 0.1 to evaluate the robustness of the primary findings. The results were consistent with the primary analysis, confirming the stability of the conclusions.

**Matching Variables and Quality**

The matching covariates included age, headache duration, accompanying symptoms (including nausea, vomiting, tinnitus, aural fullness, gait instability, and positional vertigo), and baseline scores for the Visual Analog Scale (VAS), Headache Impact Test (HIT-6), and Dizziness Handicap Inventory (DHI) together with its subscales (DHI-P, DHI-E, DHI-F). After matching, the standardized mean differences (SMDs) for most baseline variables were below 0.1, indicating excellent balance between groups. Although the SMDs for "positional vertigo" and "gait instability" were 0.17, they remained below the commonly accepted threshold of 0.2, indicating satisfactory overall matching quality (Supplementary Table S1).

**Intergroup Outcomes**

Comparative analyses at 3 months revealed that the rTMS group had significantly lower HIT-6 scores (48.08 ± 5.98 vs. 53.40 ± 5.96; P-holm < 0.05) with a large effect size (d = -0.89, 95% CI: -1.47, -0.31). Following Holm’s correction for multiple comparisons, VAS scores, total DHI score, and all DHI subscale scores showed no significant between-group differences (Supplementary Table S2).

**Within-Group Outcomes**

Both groups demonstrated significant improvements (P-holm < 0.05) from baseline to the 2-week and 3-month follow-ups in all outcome measures (VAS, HIT-6, DHI-T, and DHI subscales). The longitudinal analysis, following Holm’s correction, showed that the rTMS group exhibited sustained improvements from week 2 to month 3 in all metrics except VAS; in contrast, the control group showed this trend only for the HIT-6 score (Supplementary Table S3). However, for the DHI-T score in the rTMS group, the effect size estimate was associated with high uncertainty, as indicated by a wide confidence interval that included zero, which is more likely due to the reduced sample size under the stricter matching condition than to a true absence of effect.

**Conclusion**

This sensitivity analysis confirms the robustness of the primary conclusion: under stricter matching conditions, the rTMS group maintained significant and sustained improvements in headache-related quality of life (HIT-6) compared to the control group. No significant between-group differences were observed in headache intensity (VAS) or vestibular-related handicap (DHI).

| **Table S1: Baseline Characteristics of Patients Before and After Propensity Score Matching** | | | | | | |
| --- | --- | --- | --- | --- | --- | --- |
| **Variable** | **Before PSM** | | | | | |
|  | **Total**  **(n = 83)** | **Control group**  **(n = 41)** | **rTMS group**  **(n = 42)** | **Statistic** | **P-value** | **SMD** |
| AGE, Mean ± SD | 38.06 ± 7.36 | 38.05 ± 7.58 | 38.07 ± 7.23 | t = 0.01 | 0.99 | 0.00 |
| Duration of headache,  Mean ± SD | 4.33 ± 1.20 | 4.29 ± 1.08 | 4.36 ± 1.32 | t = 0.24 | 0.81 | 0.05 |
| VAS, M [Q1, Q3] | 5.00 [4.00, 6.00] | 5.00 [4.00, 6.00] | 5.00 [4.00, 6.00] | Z = 0.19 | 0.85 | 0.03 |
| HIT-6, M [Q1, Q3] | 65.00 [64.00, 67.00] | 65.00 [64.00, 67.00] | 66.00 [64.00, 67.00] | Z = 0.98 | 0.33 | 0.16 |
| DHI-T, M [Q1, Q3] | 32.00 [30.00, 34.00] | 32.00 [30.00, 34.00] | 32.00 [30.00, 34.00] | Z = 0.62 | 0.54 | 0.21 |
| DHI-P, M [Q1, Q3] | 10.00 [8.00, 12.00] | 10.00 [8.00, 12.00] | 10.00 [8.00, 12.00] | Z = 0.53 | 0.60 | 0.11 |
| DHI-E, M [Q1, Q3] | 10.00 [8.00, 13.00] | 10.00 [8.00, 14.00] | 10.00 [8.00, 12.00] | Z = 0.20 | 0.85 | -0.09 |
| DHI-F, M [Q1, Q3] | 10.00 [9.00, 13.00] | 10.00 [8.00, 12.00] | 12.00 [10.00, 14.00] | Z = 1.06 | 0.29 | 0.25 |
| Nausea/Vomiting, n (%) |  |  |  | χ² = 0.01 | 0.91 | -0.07 |
| 0 | 42(50.60) | 20 (48.78) | 22 (52.38) |  | 0.91 | -0.07 |
| 1 | 41 (49.40) | 21 (51.22) | 20 (47.62) |  | 0.91 | -0.07 |
| Tinnitus/Ear Fullness, n (%) |  |  |  | χ² = 0.00 | 1.00 | -0.02 |
| 0 | 51 (61.45) | 25 (60.98) | 26 (61.9) |  | 1.00 | -0.02 |
| 1 | 32 (38.55) | 16 (39.02) | 16 (38.1) |  | 1.00 | -0.02 |
| Unsteady Gait, n (%) |  |  |  | χ² = 0.02 | 0.88 | 0.09 |
| 0 | 55 (66.27) | 28 (68.29%) | 27 (64.29) |  | 0.88 | 0.09 |
| 1 | 28 (33.73) | 13 (31.71%) | 15 (35.71) |  | 0.88 | 0.09 |
| Positional Vertigo, n (%) |  |  |  | χ² = 0.38 | 0.54 | 0.19 |
| 0 | 55 (66.27) | 29 (70.73) | 26 (61.90) |  | 0.54 | 0.19 |
| 1 | 28 (33.73) | 12 (29.27) | 16 (38.10) |  | 0.54 | 0.19 |
| **Variable** | **After PSM** | | | | | |
|  | **Total (n = 50)** | **Control group (n = 25)** | **rTMS group (n = 25)** | **Statistic** | **P-value** | **SMD** |
| AGE, Mean ± SD | 38.08 ± 7.71 | 37.92 ± 7.85 | 38.24 ± 7.73 | t = 0.15 | 0.89 | 0.04 |
| Duration of headache,  Mean ± SD | 4.18 ± 1.16 | 4.24 ± 1.23 | 4.12 ± 1.09 | t = -0.36 | 0.72 | 0.10 |
| VAS, M [Q1, Q3] | 5.00 [4.00, 6.00] | 5.00 [4.00, 6.00] | 5.00 [4.00, 6.00] | Z = 0.01 | 0.99 | 0.00 |
| HIT6, M [Q1, Q3] | 65.00 [63.00, 66.00] | 65.00 [64.00, 67.00] | 65.00 [63.00, 66.00] | Z = 0.20 | 0.85 | 0.08 |
| DHI-T, M [Q1, Q3] | 32.00 [30.00, 34.00] | 32.00 [32.00, 34.00] | 32.00 [30.00, 34.00] | Z = 0.03 | 0.98 | 0.09 |
| DHI-P, Mean ± SD | 10.24 ± 2.88 | 10.16 ± 2.94 | 10.32 ± 2.87 | t = 0.20* | 0.85 | 0.06 |
| DHI-E, Mean ± SD | 11.32 ± 3.07 | 11.20 ± 3.37 | 11.44 ± 2.80 | t = 0.27* | 0.79 | 0.08 |
| DHI-F, Mean ± SD | 10.32 ± 2.47 | 10.32 ± 2.56 | 10.32 ± 2.43 | t = 0.00* | 1.00 | 0.00 |
| Nausea/Vomiting, n (%) |  |  |  | χ² = 0.00 | 1.00 | 0.08 |
| 0 | 21 (42.00) | 11 (44.00) | 10 (40.00) |  | 1.00 | 0.08 |
| 1 | 29 (58.00) | 14 (56.00) | 15 (60.00) |  | 1.00 | 0.08 |
| Tinnitus/Ear Fullness, n (%) |  |  |  | χ² = 0.00 | 1.00 | 0.00 |
| 0 | 32 (64.00) | 16 (64.00) | 16 (64.00) |  | 1.00 | 0.00 |
| 1 | 18 (36.00) | 9 (36.00) | 9 (36.00) |  | 1.00 | 0.00 |
| Unsteady Gait, n (%) |  |  |  | χ² = 0.09 | 0.76 | 0.17 |
| 0 | 34 (68.00) | 18 (72.00) | 16 (64.00) |  | 0.76 | 0.17 |
| 1 | 16 (32.00) | 7 (28.00) | 9 (36.00) |  | 0.76 | 0.17 |
| Positional Vertigo, n (%) |  |  |  | χ² = 0.09 | 0.76 | 0.17 |
| 0 | 34 (68.00) | 18 (72.00) | 16 (64.00) |  | 0.76 | 0.17 |
| 1 | 16 (32.00) | 7 (28.00) | 9 (36.00) |  | 0.76 | 0.17 |
| *Data are normally distributed after PSM. Categorical variables are presented as n (%); continuous variables are presented as Mean ± SD or Median (Q1, Q3) as appropriate. VAS, Visual Analogue Scale; HIT-6, Headache Impact Test; DHI, Dizziness Handicap Inventory; PSM, Propensity Score Matching; SMD, Standardized Mean Difference. An SMD value of < 0.1 indicates good intergroup balance. | | | | | | |

| **Table S2. Comparison of Clinical Outcomes Between the Control and rTMS Groups** | | | | | | |
| --- | --- | --- | --- | --- | --- | --- |
| **Variable** | **Time Point** | **Control group**  **(n = 25)** | **rTMS group**  **(n = 25)** | **Statistic** | **P-value (Holm-corrected)** | **Effect Size (95%CI)** |
| VAS | baseline | 5.00 [4.00, 6.00] | 5.00 [4.00, 6.00] | Z = -0.01 | 1.00 | r = 0.00 (-0.28, 0.28) |
|  | 2 weeks | 4.00 [4.00, 5.00] | 4.00 [4.00, 5.00] | Z = 0.00 | 1.00 | r = 0.00 (-0.28, 0.28) |
|  | 3 months | 4.00 [4.00, 5.00] | 4.00 [4.00, 4.00] | Z = -1.30 | 0.58 | r = -0.18 (-0.09, 0.45) |
| HIT-6 | baseline | 65.00 [64.00, 67.00] | 65.00 [63.00, 66.00] | Z = -0.20 | 1.00 | r = 0.03 (-0.25, 0.31) |
|  | 2 weeks | 58.00 [56.00, 60.00] | 59.00 [56.00, 60.00] | Z = -0.58 | 1.00 | r = 0.08 (-0.20, 0.36) |
|  | 3 months | 53.40 ± 5.96 | 48.08 ± 5.98 | t = -3.15* | <0.05 | d = -0.89 (-1.47, -0.31) |
| DHI-T | baseline | 32.00 [32.00, 34.00] | 32.00 [30.00, 34.00] | Z = -0.03 | 0.98 | r = -0.00 (-0.28, 0.28) |
|  | 2 weeks | 26.96 ± 2.95 | 27.76 ± 3.02 | t = 0.95* | 0.70 | d = 0.27 (-0.29, 0.83) |
|  | 3 months | 26.00 [24.00, 28.00] | 24.00 [22.00, 26.00] | Z = -2.37 | 0.053 | r = 0.34 (0.09, 0.58) |
| DHI-P | baseline | 10.16 ± 2.94 | 10.32 ± 2.87 | t = 0.19* | 1.00 | d = 0.06 (-0.50, 0.61) |
|  | 2 weeks | 7.68 ± 2.81 | 8.32 ± 2.75 | t = 0.81* | 1.00 | d = 0.23 (-0.33, 0.79) |
|  | 3 months | 8.00 [6.00, 10.00] | 8.00 [6.00, 8.00] | Z = -0.13 | 1.00 | r = 0.02 (-0.26, 0.30) |
| DHI-E | baseline | 11.20 ± 3.37 | 11.44 ± 2.80 | t = 0.27* | 1.00 | d = 0.08 (-0.48, 0.63) |
|  | 2 weeks | 10.00 [8.00, 12.00] | 10.00 [8.00, 12.00] | Z = -0.17 | 1.00 | r = 0.02 (-0.26, 0.30) |
|  | 3 months | 8.00 [8.00, 12.00] | 8.00 [8.00, 10.00] | Z = -1.57 | 0.35 | r = 0.22 (-0.04, 0.49) |
| DHI-F | baseline | 10.32 ± 2.56 | 10.32 ± 2.43 | t = 0.00* | 1.00 | d = 0.00 (-0.55, 0.55) |
|  | 2 weeks | 10.00 [8.00, 10.00] | 10.00 [8.00, 12.00] | Z = -0.01 | 1.00 | r = 0.00 (-0.28, 0.28) |
|  | 3 months | 10.00 [8.00, 10.00] | 8.00 [8.00, 10.00] | Z = -1.19 | 0.70 | r = 0.17 (-0.10, 0.44) |
| *Data are normally distributed. P-values were adjusted for multiple comparisons using the Holm method. Effect sizes are presented as Cohen's d for parametric tests or r (Z/√N) for non-parametric tests, with 95% Confidence Intervals. | | | | | | |

| **​** **Table S3. Longitudinal Comparisons of Clinical Outcomes Within the Control and rTMS Groups** | | | | | | | |
| --- | --- | --- | --- | --- | --- | --- | --- |
| **Variable** | **Comparison** | **Control group(n=25)** | | | **rTMS group(n=25)** | | |
|  |  | **Statistic** | **P-value (Holm-corrected)** | **Effect Size (95%CI)** | **Statistic** | **P-value (Holm-corrected)** | **Effect Size (95%CI)** |
| VAS | baseline vs. 2 weeks | Z = 2.46 | 0.01 | r = 0.49 (0.19, 0.80) | Z = 2.54 | <0.01 | r = 0.51 (0.21, 0.80) |
|  | baseline vs. 3 months | Z = 2.88 | 0.03 | r = 0.58 (0.31, 0.84) | Z = 2.97 | 0.02 | r = 0.60 (0.35, 0.86) |
|  | 2 weeks vs. 3 months | Z = 0.36 | 0.72 | r = 0.07 (-0.33, 0.47) | Z = 1.63 | 0.10 | r = 0.33 (-0.03, 0.68) |
| HIT-6 | baseline vs. 2 weeks | Z = 4.10 | <0.001 | r = 0.82 (0.69, 0.95) | Z = 4.37 | <0.001 | r = 0.87 (0.78, 0.97) |
|  | baseline vs. 3 months | Z = 4.36 | <0.001 | r = 0.87 (0.78, 0.97) | Z = 4.37 | <0.001 | r = 0.87 (0.78, 0.97) |
|  | 2 weeks vs. 3 months | Z = 3.53 | <0.001 | r = 0.71 (0.50, 0.91) | Z = 4.10 | <0.001 | r = 0.82 (0.69, 0.95) |
| DHI-T | baseline vs. 2 weeks | Z = 4.34 | <0.001 | r = 0.87 (0.77, 0.97) | t = 7.24* | <0.001 | d = 1.45 (-0.22, 3.12) |
|  | baseline vs. 3 months | Z = 4.22 | <0.001 | r = 0.84 (0.73, 0.96) | t = 9.61* | <0.001 | d = 1.92 (-0.83, 4.67) |
|  | 2 weeks vs. 3 months | Z = 1.29 | 0.20 | r = 0.26 (-0.11, 0.63) | t = 5.75* | <0.001 | d = 1.15 (-0.47, 2.77) |
| DHI-P | baseline vs. 2 weeks | Z = 4.65 | <0.001 | r = 0.93 (0.88, 0.98) | Z = 4.50 | <0.001 | r = 0.90 (0.83, 0.98) |
|  | baseline vs. 3 months | Z = 4.12 | <0.001 | r = 0.82 (0.70, 0.95) | Z = 4.12 | <0.001 | r = 0.82 (0.69, 0.95) |
|  | 2 weeks vs. 3 months | Z = 0.25 | 0.80 | r = 0.05 (-0.35, 0.45) | Z = 2.01 | 0.04 | r = 0.40 (0.07, 0.74) |
| DHI-E | baseline vs. 2 weeks | Z = 2.46 | 0.02 | r = 0.49 (0.19, 0.80) | Z = 2.97 | <0.01 | r = 0.59 (0.33, 0.85) |
|  | baseline vs. 3 months | Z = 2.81 | 0.03 | r = 0.56 (0.28, 0.83) | Z = 3.74 | <0.001 | r = 0.75 (0.57, 0.92) |
|  | 2 weeks vs. 3 months | Z = 0.92 | 0.36 | r = 0.18 (-0.20, 0.57) | Z = 2.88 | <0.01 | r = 0.58 (0.31, 0.84) |
| DHI-F | baseline vs. 2 weeks | Z = 2.88 | <0.01 | r = 0.58 (0.32, 0.85) | Z = 2.35 | 0.04 | r = 0.47 (0.16, 0.78) |
|  | baseline vs. 3 months | Z = 3.09 | <0.01 | r = 0.62 (0.37, 0.86) | Z = 3.09 | <0.01 | r = 0.62 (0.37, 0.86) |
|  | 2 weeks vs. 3 months | Z = 0.94 | 0.35 | r = 0.19 (-0.20, 0.57) | Z = 2.37 | 0.04 | r = 0.47 (0.16, 0.78) |
| *Data are normally distributed. P-values were adjusted for multiple comparisons using the Holm method. Effect sizes are presented as Cohen's d for parametric tests or r (Z/√N) for non-parametric tests, with 95% Confidence Intervals. | | | | | | | |
